# Optimal RNA isolation method and primer design to detect gene knockdown by qPCR when validating *Drosophila* transgenic RNAi lines

**DOI:** 10.1186/s13104-017-2959-0

**Published:** 2017-11-29

**Authors:** Roslyn L. Mainland, Taylor A. Lyons, Mike M. Ruth, Jamie M. Kramer

**Affiliations:** 1 Department of Physiology and Pharmacology, Schulich School of Medicine and Dentistry, Western University, 1151 Richmond St., London, ON N6A 5C1 Canada; 2 Department of Biology, Faculty of Science, Western University, London, ON Canada; 3 Division of Genetics and Development, Children’s Health Research Institute, London, ON Canada

**Keywords:** RNAi knockdown efficiency, Quantitative real-time PCR, UAS-Gal4 system, *Drosophila* transgenic RNAi lines

## Abstract

**Objective:**

RNA interference is employed extensively in *Drosophila* research to study gene function within a specific cell-type or tissue. Thousands of transgenic *Drosophila* lines have been generated to express double stranded RNA for gene knockdown; however, no standardized method exists for quantifying their knockdown efficiency. Since antibodies are not available for many proteins, quantitative real-time PCR is often used. Here, we explore how primer design and RNA isolation method can influence detection of gene knockdown using qPCR.

**Results:**

We tested differences in detected gene knockdown efficiency when using purified polyadenylated mRNA or total RNA as templates for cDNA synthesis. We also tested two different primer locations for each gene: one to amplify a region 5′ of the RNAi cut site, and one to amplify a region 3′ of the cut site. Consistently, the strongest gene knockdown was detected when qPCR was performed using 5′ primer sets in combination with mRNA-derived cDNA. Our results indicate that detection of undegraded mRNA cleavage fragments can result in underestimation of true knockdown efficiency for a RNAi construct. Purification of polyadenylated mRNA, combined with primers designed to amplify the non-polyadenylated 5′ mRNA cleavage fragment can avoid this problem.

**Electronic supplementary material:**

The online version of this article (10.1186/s13104-017-2959-0) contains supplementary material, which is available to authorized users.

## Introduction

RNA interference (RNAi) is an important technique for performing loss-of-function experiments in both cell culture and in vivo models. RNAi-induced gene knockdown can give insight into the function of a gene and its encoded protein, within the context of a specific cell-type or tissue. The endogenous RNAi pathway is triggered when a double stranded RNA (dsRNA) molecule is present within a cell [1]. The Dicer-2 protein cleaves dsRNA into short interfering RNA (siRNA) fragments. Argonaute and the RNA-induced silencing complex associate with these siRNA fragments to cleave target mRNA at a sequence-specific site. This cleavage is directed by the complementarity of the siRNA to the mRNA, leaving 5′ and 3′ mRNA fragments that are eventually degraded, and do not contribute to protein production. Current evidence suggests that the 5′ fragment is degraded through a nonstop mRNA decay pathway, while the 3′ fragment is degraded by cellular 5′ to 3′ exonucleases [2, 3].

RNAi is employed extensively in *Drosophila* research through use of the UAS-Gal4 binary transgenic expression system [4]. Thousands of transgenic lines are available that express dsRNA hairpins under the control of the yeast upstream activation sequence (UAS) enhancer [5, 6]. When these *UAS*-*RNAi* lines are crossed to a Gal4 ‘driver’ line, the expression of dsRNA leads to activation of cellular RNAi machinery, which can induce tissue-specific knockdown of a given gene. This system has become an important tool for investigating gene function in specific tissues within the context of a whole organism. Despite the importance and widespread use of *Drosophila* RNAi reagents, there are still several challenges and limitations to consider. RNAi-mediated gene knockdown is not 100% efficient and knockdown levels can vary considerably between various RNAi lines [7]. This can lead to inconsistencies in phenotypic effects for different RNAi lines that target the same gene. Therefore, gene knockdown should be quantified for different RNAi lines. Western blotting is an ideal technique for verifying gene knockdown; however, this method is not effective for tissue-specific RNAi knockdown. In these cases, one must rely on immunohistochemistry, which is only partially quantitative. Furthermore, specific and effective antibodies are often not available for proteins of interest. They are also expensive and sometimes difficult to generate. Northern blotting is a possibility to detect mRNA knockdown, however, similar to Western blotting it is not useful for tissue specific knockdown, and many labs do not have the facilities to work with radioactive labeled probes. Real-time quantitative PCR (qPCR) can be used as an antibody- and probe-independent method to quantify gene knockdown efficiency, and has the advantage that is more sensitive than both Western and Northern blotting.

In the *Drosophila* community, considerable effort has been made in the development of a qPCR primer design resource (FlyPrimerBank) that considers the optimal primer sequence, as well as the location of primer amplicons with respect to RNAi reagents [8]. However, there is no standard protocol for quantifying RNAi knockdown, and most RNAi reagents have yet to be tested.

**Fig. 1:**
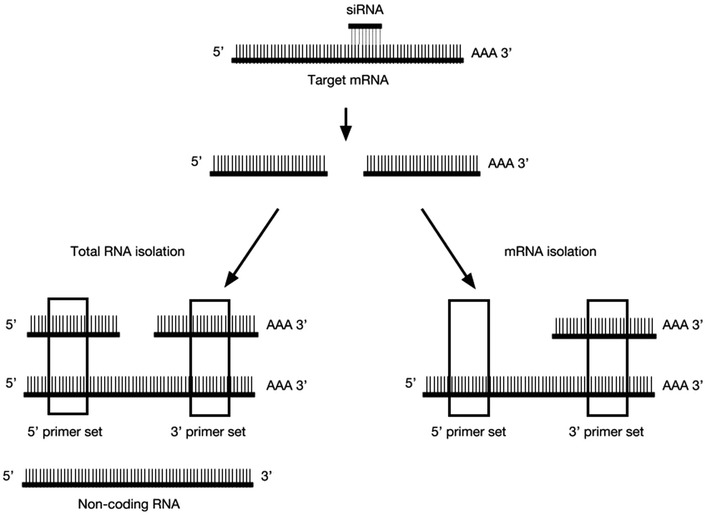
Schematic representation of the experimental setup. siRNAs direct site-specific cleavage of mRNAs, resulting in a 5′ and 3′ mRNA cleavage fragments. After RNA isolation total RNA samples consist of uncleaved mRNA transcripts and non-coding RNA, as well as undegraded 5′ and 3′ mRNA cleavage fragments. Purification of mRNA using poly-T beads excludes 5′ mRNA cleavage fragments and non-coding RNAs that are not polyadenylated. As indicated by the boxes, 5′ and 3′ primer sets could detect different species of RNA depending on the isolation method

Here, we identify optimal qPCR conditions for testing gene knockdown efficiency in combination with the UAS-Gal4 driven *Drosophila* RNAi system. Specifically, we identify important factors relating to RNA isolation method and primer amplicon position relative to the RNAi target site on the mRNA. Previous studies performed on cultured cells have reported discrepancies between Western blotting results, which detect protein levels, and qPCR measurement, which detects mRNA levels. In these cases, there is a clear loss of protein that is not reflected by quantification of mRNA [9, 10]. Holmes et al. tested mRNA knockdown detection using several primer sets spanning a single gene and found that 3′ primer sets were not able to detect siRNA mediated knockdown as well as 5′ primer sets [9]. They suggested that this was due to the persistence of the 3′ mRNA cleavage fragment in the single gene that was tested [9]. Detection of the 3′ cleavage fragment would result in overestimation of the amount of functional mRNA present in the cell. We expand upon the study by Holmes et al. by testing several loci using primer sets that amplify the 5′ fragment of cleaved mRNA, in combination with cDNA synthesized from purified polyadenylated mRNA. In principle, only uncleaved functional mRNA transcripts would be detected under these conditions (Fig. 1). By testing a combination of different primer locations and RNA isolation methods at multiple loci, we were able confirm the untested theory first proposed by Holmes et al. [9], and provide optimal conditions for testing RNAi mediated knockdown using transgenic *Drosophila* RNAi lines.

## Main text

**Fig. 2:**
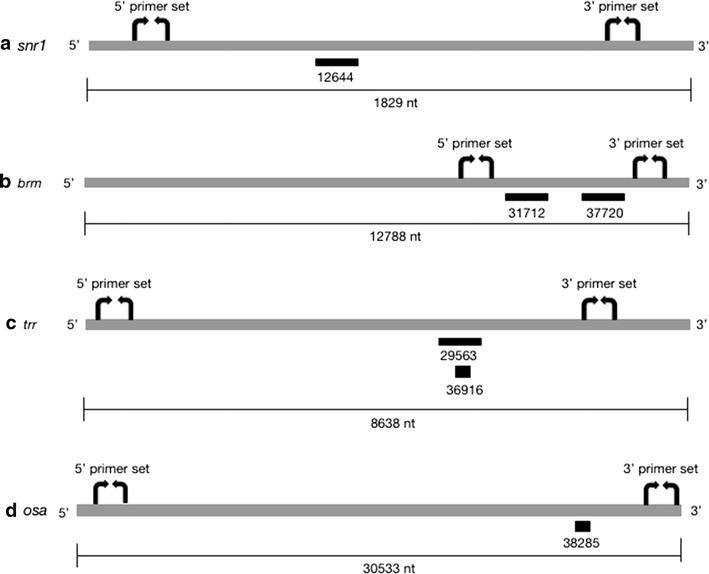
Location of RNAi cut sites and primer amplicons. Schematic representation of *trr* (**a**), *brm* (**b**), *osa* (**c**) and *snr1* (**d**) genes, showing siRNA cut sites and location of designed primer sets. siRNAs are indicated by black bars with the *UAS*-*RNAi* stock number listed below. Primer sets are represented by curved arrows. Drawings are approximately to scale and mRNA length in nucleotides (nt) is indicated

Expression of dsRNA using the UAS-Gal4 system activates endogenous cellular RNAi machinery. This leads to cleavage of target mRNA molecules at a specific site, producing 5′ and 3′ mRNA fragments that are eventually degraded (Fig. 1). Only uncleaved mRNA transcripts contribute to protein production; however, mRNA cleavage products that have yet to be degraded are likely represented in cDNA libraries that are used as a template for qPCR. Detection of these fragments would result in overestimation of the amount of functional mRNA present in the cell. The use of primer sets that amplify the 5′ fragment of cleaved mRNA in combination with cDNA synthesized from purified polyadenylated mRNA should circumvent this problem, allowing only for the detection of uncleaved mRNA transcripts (Fig. 1). To test this idea, we performed qPCR using two different primer sets—one that amplified a region on the mRNA transcript 5′ of the siRNA cut site, and the other that amplified from the 3′ mRNA cleavage fragment (Figs. 1, 2). We performed qPCR on cDNA synthesized from total RNA, which contains mRNA transcripts, non-coding RNA, and siRNA-mediated cleavage products, and compared this to qPCR results using cDNA derived from mRNA purified using poly-T beads, which excludes 5′ mRNA cleavage fragments (Fig. 1).

### Fly stocks, housing, larva collection

*Drosophila UAS*-*RNAi* strains were obtained from the Bloomington *Drosophila* Stock Center (*trr*-FBgn0023518, *UAS*-*trr* ^29563^ and *UAS*-*trr* ^36916^; *osa*-FBgn0261885, *UAS*-*osa* ^*38285*^; *brm*-FBgn0000212, *UAS*-*brm* ^37720^ and *UAS*-*brm* ^31712^) and the Vienna Drosophila Resource Center (VDRC) (*snr1*-FBgn0011715, *UAS*-*snr1* ^*12644*^). All experiments were performed on standard fly food at 25 °C in 70% humidity on a 12-h light/dark cycle. RNAi stocks were crossed to an in-house *UAS*-*Act*-*Gal4/CyO*-*ActGFP* driver line, created by balancing y; *Act*-*Gal4*/CyO (Bloomington 25374) to a *CyO*-*ActGFP* balancer (Bloomington 4533). As a control, this driver line was also crossed to a *UAS*-*mCherry*-*RNAi* line (Bloomington 35785). Third instar larvae containing *Act*-*Gal4* and the *UAS*-*RNAi* were selected using a Nightsea fluorescence adaptor (Nightsea, Cat. No SFA-LFS-RB) based on the absence of GFP. Ten larvae of each genotype were pooled into a single tube and flash frozen in liquid nitrogen, followed by storage at − 80 °C. Three biological replicates of each genotype were collected.

### Primer design and selection

Two primer sets were designed for each targeted gene: one to amplify a region 5′ of the siRNA cut site on the target mRNA, and the other to amplify a 3′ region (Fig. 2, Additional file 1: Table S1). Primers were selected from FlyPrimerBank [10], if available, or designed using Primer3 v.4.1.0 [11, 12]. Primers were ordered commercially and validated for efficiency using a cDNA dilution series, according the equation “efficiency = 10^[− 1/slope]^” [13].

### Total RNA isolation, mRNA isolation, and cDNA synthesis

Larvae were disrupted in QIAzol lysis reagent (Qiagen, Cat. No. 79306) using a pestle and further homogenized using QIAshredder tissue homogenization columns (Qiagen, Cat. No. 79654). Total RNA was isolated using the Qiagen RNeasy Lipid Tissue Mini Kit (Qiagen, Cat. No. 74804) according to the manufacturer’s protocol with on-column DNase digestion (Qiagen, Cat. No. 79254). cDNA from total RNA was synthesized using the SensiFast cDNA Synthesis Kit (Bioline, Cat. No. BIO-65053). Remaining total RNA was used for mRNA isolation using the Oligotex mRNA Mini Kit (Qiagen, Cat. No. 70022) scaled to < 0.25 mg. Samples were eluted twice in 20 μL of Buffer OEB, and cDNA was synthesized using the SensiFast cDNA Synthesis Kit.

### Real-time quantitative polymerase chain reaction

Quantitative PCR assays were conducted using the SensiFAST SYBR No-Rox kit (Bioline, Cat. No. BIO-98020). Reaction mixtures were composed according to the manufacturer’s protocol. Reactions were carried out in a Bio-Rad CFX384 Real-Time System using the following cycling conditions: 95 °C for 2 min, followed by 40 cycles at 95 °C for 5 s and 65 °C for 30 s. Three qPCR technical replicates were conducted for each biological replicate. Relative expression was normalized to two reference genes, *eIF2Bγ* (FBgn0034029) and *βCOP* (FBgn0008635).

### Statistics

Gene expression was normalized to two reference genes (*eIF2Bγ* and *βCOP*) using the ∆∆Ct method, correcting for primer efficiencies according to the method described by Pfaffl [13]. One-tailed t tests were performed using Microsoft Excel (version 15.37) to determine if there was a significant reduction in mRNA level detected for each condition, compared to the *UAS*-*mCherry*-*RNAi* control. To compare differences in mRNA levels between primer set locations and RNA isolation methods, GraphPad Prism 7 was used to perform two-way ANOVA on gene expression values normalized to the *UAS*-*mCherry*-*RNAi* control, with false discovery rate corrections for multiple comparisons.

## Results

We expressed *Drosophila UAS*-*RNAi* transgenes (*UAS*-*snr1* ^*12644*^, *UAS*-*brm* ^*31712*^, *UAS*-*brm* ^*37720*^, *UAS*-*trr* ^*29563*^, *UAS*-*trr* ^*36916*^ and *UAS*-*osa* ^*38285*^) using the ubiquitous *Actin*-*Gal4* driver line. For all of these lines, *Act*-*Gal4* mediated expression resulted in lethality at the pupal stage, consistent with the function of these genes, which are known to be essential for normal development [14]. This suggests that these RNAi lines induce a strong enough knockdown to produce biological effects, and is congruous with previous studies that suggest the effectiveness of these lines in inducing gene knockdown [15–19].

**Fig. 3:**
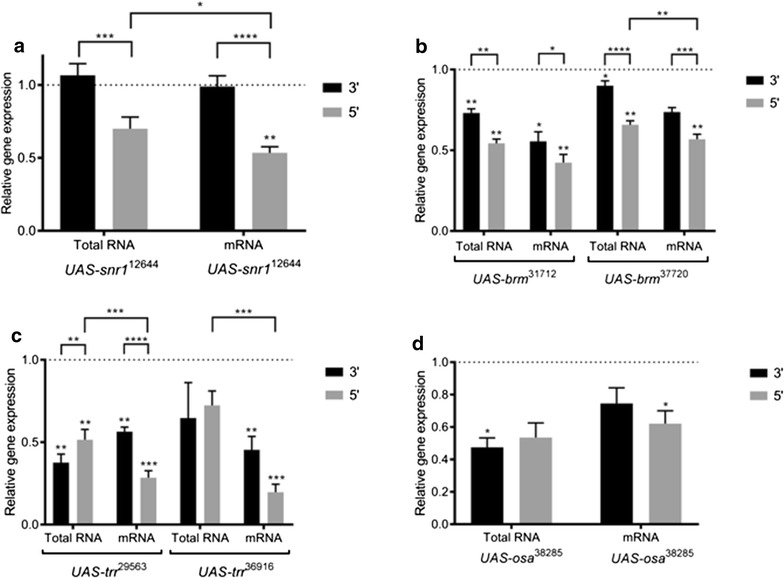
Primer location and RNA isolation method affect qPCR knockdown detection. qPCR was conducted on cDNA synthesized from total RNA samples and mRNA samples. Two primer sets—one amplifying 5′ of the siRNA cut site, the other amplifying 3′ of the siRNA cut site—were compared. Relative gene expression of **a** *snr1*, **b** *brm*, **c** *osa* and **d** *trr* was measured by qPCR in third instar larvae after ubiquitous expression of *UAS*-*RNAi* constructs with *Act*-*Gal4*. Expression levels were normalized to the reference genes, *eIF2Bγ* and *βCOP*. Shown here, are relative expression values compared to the *UAS*-*mCherry*-*RNAi* control (indicated by the dotted line). Asterisks directly above bars indicate a significant knockdown compared to the control, while asterisks above brackets indicate significant differences in gene expression between different conditions—total RNA vs. mRNA, 3′ vs. 5′ primer set (*p < 0.05, **p < 0.01, ***p < 0.001, ****p < 0.0001). Error bars indicate the standard error of the mean

Despite the lethal phenotype upon expression of *UAS*-*snr1* ^*12644*^ with *Act*-*Gal4*, significant knockdown was only detected when using the 5′ *snr1* primer set in combination with purified polyadenylated mRNA (Fig. 3a). For all other conditions—3′ primers, and 5′ primer with total RNA—detected mRNA levels were not significantly different from the control. Furthermore, the 5′ primer set detected a significantly greater knockdown on cDNA derived from purified mRNA (relative expression = 53.4%), as compared to total RNA (70.1%). In keeping with our model (Fig. 1), this result suggests that RNAi cleavage fragments may be masking the true RNAi knockdown efficiency, which could have a serious impact on interpretation of data and planning of experiments.

For both *UAS*-*brm* ^37720^ and *UAS*-*brm* ^31712^, a significant knockdown was detected for nearly all conditions tested when compared to controls (Fig. 3b). However, there were also significant differences in the level of knockdown between the different conditions. 5′ primer sets consistently detected a greater *brm* knockdown than 3′ primer sets. For both *brm* RNAi lines tested the strongest knockdown was observed using purified polyadenylated mRNA in combination with a 5′ primer set (56.8% for *UAS*-*brm* ^37720^ and 42.4% for *UAS*-*brm* ^31712^), while the weakest knockdown was observed when using 3′ primer sets in combination with total RNA (90.1% for *UAS*-*brm* ^37720^ and 73.2% for *UAS*-*brm* ^31712^). Again, these results suggest that RNAi cleavage fragments may be masking knockdown under suboptimal testing conditions.

For *UAS*-*trr* ^29563^ and *UAS*-*trr* ^36916^, we again observed the strongest detectable knockdown when using 5′ primers in combination with purified mRNA (Fig. 3c). This was especially striking for *UAS*-*trr* ^36916^, which showed almost threefold greater knockdown when using 5′ primers and mRNA (relative expression = 20.0%) compared to when total RNA was used (72.4 and 64.7% using 5′ and 3′ primer sets, respectively).

Finally, for one RNAi line tested, *UAS*-*osa* ^38285^, there was no significant difference in knockdown detection between the different primer set locations and RNA templates (Fig. 3d), suggesting that these factors are not always critical for optimal knockdown detection.

The data presented in this study suggests that detecting RNAi-mediated knockdown using qPCR is dependent on amplicon location, as well as RNA isolation technique. Our results indicate that the best practice for validating RNA-mediated gene knockdown using qPCR is to use cDNA synthesized from purified polyadenylated mRNA, in combination with a primer set that amplifies from the non-polyadenylated 5′ mRNA cleavage product. This approach ensures that mRNA cleavage products will not be detected by qPCR, providing an accurate estimate of functional mRNA levels.

## Limitations

The degree to which non-functional mRNA cleavage fragments impact knockdown detection is quite variable between different RNAi lines tested. Having only tested six RNAi lines, we cannot predict how universal this phenomenon is, and the mechanisms causing this variation are not clear.

It is also important to consider the limitations of using qPCR to represent protein levels, as mRNA abundance does not always correlate to protein levels [10, 20, 21].

cDNA synthesis, a necessary step before performing qPCR, can also result in potential bias and non-uniform representation of mRNA [22, 23]. In this study we attempted to overcome this limitation by using the Sensifast cDNA synthesis kit, which blends random hexamer primers and anchored oligo dT (deoxythymine) to result in unbiased 3′ and 5′ coverage and reverse transcription of all gene regions.

## Additional file

**Additional file 1: Table S1.** Sequences of RT-qPCR primers used to detect gene knockdown.

## Abbreviations

dsRNA: double stranded RNA
dT: deoxythymine
qPCR: real-time quantitative polymerase chain reaction
RNAi: RNA interference
siRNA: short interfering RNA
UAS: upstream activation sequence
VDRC: Vienna Drosophila Resource Centre
