# Supplementary material for: Optimal RNA isolation method and primer design to detect gene knockdown by qPCR when validating Drosophila transgenic RNAi lines
Source: BMC Res Notes. 2017 Nov 29;10:647. doi: 10.1186/s13104-017-2959-0 (PMC5707822; doi:10.1186/s13104-017-2959-0)
Supplement: Supplementary file 1 — Additional file 1: Table S1. Sequences of RT-qPCR primers used to detect gene knockdown. [file 13104_2017_2959_MOESM1_ESM.docx]

**Table S1.** Sequences of RT-qPCR primers used to detect gene knockdown.

| Target | Location on Gene | Forward Primer Sequence | Reverse Primer Sequence |
| --- | --- | --- | --- |
| *trr* | 5’ of RNAi cut site | GAACGCCTATTGGAGCTACTAC | CGGATTTCTGTGTGAGGATTGT |
| *trr* | 3’ of RNAi cut site | AGCAAGCAGTTCGTGCATTC | CGCCAAGTAGACGTTGTTGC |
| *snr1* | 5’ of RNAi cut site | CTACTACGTGGGCTCGGAAG | AGCGATACAGAGCTGGCTAAG |
| *snr1* | 3’ of RNAi cut site | GCCGAAATGGAGAAGAAAATC | CCAACCAGTTGTGGTATTGG |
| *osa* | 5’ of RNAi cut site | AATGTTGCCCCACATCCATAC | GTGCAGATGATGATAGTGCATGA |
| *osa* | 3’ of RNAi cut site | GCTGCGAAAATTCCAACTTC | TGTTTGCTTGCCTTCATCTG |
| *brm* | 5’ of RNAi cut site | TGCACGAGACGAGATACCAC | GCTTTCGTTCAGCCTCTAGC |
| *brm* | 3’ of RNAi cut site | GCGACGAAAGCGTAAGAATC | TGTGCTTGATAACCGCACTC |
| *ßCOP* |  | AGCGGGTAATCAAGTTGCTG | GGCAGGACGAAGCGTATGA |
| *eiF2Bγ* |  | GACCTCCTAAAACGGTGCAG | TTCTGGTCCAGAATGGTGG |
